# *Lactiplantibacillus plantarum* HY7715 Alleviates Restraint Stress-Induced Anxiety-like Behaviors by Modulating Oxidative Stress, Apoptosis, and Mitochondrial Function

**DOI:** 10.3390/ijms26189251

**Published:** 2025-09-22

**Authors:** Kippuem Lee, Daehyeop Lee, Haeryn Jeong, Joo Yun Kim, Jae Jung Shim, Jae Hwan Lee

**Affiliations:** R&BD Center, Hy Co., Ltd., 22 Giheungdanji-ro 24 Beon-gil, Giheung-gu, Yongin-si 17086, Republic of Korea; joy4917@hanmail.net (K.L.); flywhy7@hy.co.kr (D.L.); haeryn@hy.co.kr (H.J.); jjshim@hy.co.kr (J.J.S.); jaehwan@hy.co.kr (J.H.L.)

**Keywords:** anxiety, *Lactiplantibacillus plantarum* HY7715, neuroprotection, psychobiotics

## Abstract

Anxiety disorders are closely associated with oxidative stress-mediated neuronal damage, mitochondrial dysfunction, and apoptosis. In this study, we investigated the neuroprotective effects of *Lactiplantibacillus plantarum* HY7715 in a mouse model of restraint stress-induced anxiety, and in neuronal cell models (HT-22 mouse hippocampal neuroblast cell and SH-SY5Y human neuroblastoma cells). Oral administration of HY7715 (1 × 10^9^ CFU/kg/day) alleviated anxiety-like behaviors significantly, as shown by increased central exploration in the open field test and prolonged open-arm activity in the elevated plus maze. HY7715 reduced serum norepinephrine levels elevated by stress, and restored hippocampal expression of brain-derived neurotrophic factor, while suppressing pro-inflammatory (*NF-κB*, *IL-6*) and pro-apoptotic (*BAX*, *caspase-3*) markers. It also increased expression of mitochondrial regulatory genes (*SIRT1*, *mTOR*), and decreased that of cytochrome c, in brain tissue. Histological analysis revealed that HY7715 preserved neuronal integrity in the CA1 and CA3 hippocampal regions. In vitro, HY7715 attenuated oxidative stress-induced cytotoxicity, decreased intracellular ROS accumulation, maintained mitochondrial activity, and inhibited apoptosis of both neuronal cell types, showing greater efficacy than the strain type *L. plantarum* KCTC3108. These findings suggest that HY7715 exerts neuroprotective effects by modulating oxidative stress/apoptosis/mitochondrial pathways, and highlight its potential as a psychobiotic for stress-related neuropsychiatric disorders.

## 1. Introduction

Oxidative stress plays a key role in the pathogenesis of several brain-related diseases, including cognitive decline, dementia, and Parkinson’s disease [1]. In neurological diseases, oxidative stress can disrupt defense mechanisms and cause neuronal damage [2]. Oxidative stress is a process in which imbalance between reactive oxygen species (ROS) production and antioxidant defense mechanisms causes excessive oxidative damage to cellular components and impairs the structure and function of neurons [3]. This imbalance plays a significant role in cellular and organismic aging [4]. Neurons in the brain are particularly vulnerable to oxidative stress because of their high oxygen demand, abundance of redox-active metals, and high levels of polyunsaturated fatty acids [5]. In this regard, recent studies in various behavioral models report that oxidative stress is also associated with anxiety [6,7]. Excessive exposure to stress in animals can induce physiological and behavioral cognitive changes such as anxiety, depression, and other neuropsychiatric disorders [8]. Furthermore, development of these stress-related disorders may involve various complex systems, including the limbic system and the hypothalamic–pituitary–adrenal (HPA) axis [9]. Also, exposure of the sympathetic-adreno-medullar axis to stress results in secretion of norepinephrine, which is an adrenergic neurotransmitter, thereby activating the sympathetic nervous system and causing a state of alertness [10]. In addition, norepinephrine increases ROS, which cause cell damage and promote oxidative stress and inflammatory responses [11]. In addition, excess intracellular levels of ROS damage proteins, nucleic acids, lipids, membranes and organelles, which can in turn increase activation of cell death processes [12]. In particular, apoptosis is triggered when cells are damaged by oxidative stress [13,14]. When stress increases ROS levels in brain cells, cytochrome c, caspase 3, and caspase 9 are activated to trigger apoptosis. This can increase the rate of DNA and protein damage in neurons [15,16]. Additionally, ROS play an important role in mitochondrial function, release of mitochondrial death amplifier factor, activation of intracellular caspase, and DNA damage [17]. Clinical studies have also reported that patients with anxiety and stress-related disorders show elevated oxidative stress markers, such as malondialdehyde and 8-hydroxy-2′-deoxyguanosine (8-OHdG), together with reduced antioxidant defense capacity. These findings complement preclinical data indicating that oxidative stress not only contributes to neuronal apoptosis but also worsens mitochondrial dysfunction under chronic stress conditions. Nevertheless, the causal role of oxidative stress in psychiatric symptoms remains unclear, highlighting the importance of mechanistic studies that can bridge clinical observations and experimental models [7,18].

Probiotics are live microorganisms that, when consumed in adequate amounts, provide health benefits to the host, including gut health, immune enhancement, and weight management [19]. Lactic acid bacteria (LAB) are the most widely studied genus of bacteria, and their probiotic properties are well known [20]. The health benefits of certain fermented foods, which are generally considered to be rich sources of LAB, have been known for thousands of years [21]. Representative examples include *Lactobacillus rhamnosus JB-1*, which alleviates anxiety- and depression-like behaviors in rodent models, and *Bifidobacterium longum 1714*, which has demonstrated stress-relieving effects in human clinical trials. Recent studies, including both preclinical animal experiments and human clinical trials, report that probiotics exert beneficial effects on the central nervous system by regulating the gut–brain axis, as well as modulating the incidence of mental disorders such as depression, anxiety, and Alzheimer’s disease [22,23,24,25]. These studies suggest that probiotics may affect brain function and behavior by promoting neurotransmitter production or reducing oxidative stress in the brain [26]. *Lactiplantibacillus plantarum* HY7715 (HY7715), a species of LAB, is able to colonize the human gastrointestinal tract successfully. According to our previous studies, HY7715 (derived from kimchi, a traditional Korean food) can trigger overproduction of vitamin B2 [27]. Furthermore, both live and dead HY7715 strains improve age-related sarcopenia. In addition, we demonstrated previously that HY7715 produces specific functional extracellular vesicles, which mediate various health benefits [28,29]. These data suggest that HY7715 may have beneficial effects on several age-related diseases; however, it is unclear whether consumption of HY7715 probiotics improves brain function and mental health through the gut–brain axis. Therefore, we conducted both in vitro and in vivo studies using a mouse model of restraint stress-induced anxiety and neuronal cell models. The aim of the present study was to evaluate the neuroprotective effects of *Lactiplantibacillus plantarum* HY7715 against oxidative stress-mediated neuronal damage and to elucidate its underlying mechanisms, with a particular focus on oxidative stress, apoptosis, and mitochondrial function.

## 2. Results

### 2.1. HY7715 Alters Expression of Stress Response Markers in Restraint-Stressed Mice

**Figure 1:**
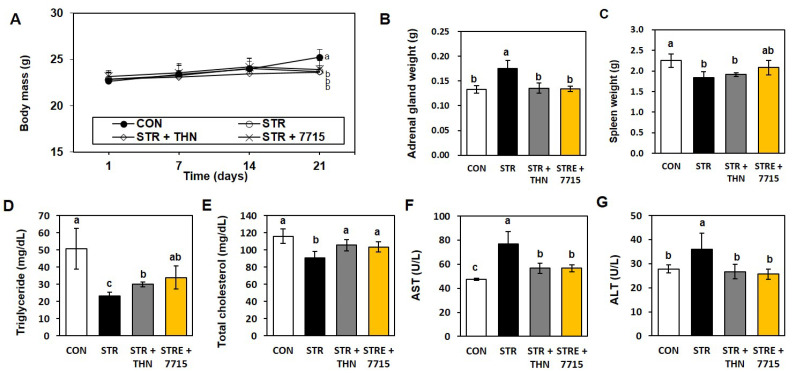
The body mass, tissue weight, and biochemical parameters of restraint-stressed mice, Body mass (**A**), adrenal gland weight (**B**), and spleen weight (**C**) were measured. Serum parameters, including triglyceride (**D**), total cholesterol (**E**), aspartate aminotransferase (**F**), and (**G**) alanine aminotransferase (ALT), were measured (*n* = 5). Statistics data were analyzed using one-way ANOVA followed by Tukey’s post hoc test. Different letters present significant differences (*p* < 0.05). CTR, control mice; STR, stress-exposed mice; STR + TFN, stress-exposed mice treated with 50 mg/kg/day L-theanine; STRE + 7715, stress-exposed mice treated with HY7715 probiotics (10^8^ CFU/kg/day).

The average body weight (23.7 ± 0.5 g) of mice exposed to restraint stress was significantly lower than that of the control group (25.2 ± 0.8 g); however, HY7715 had any significant effect on the body weight of stressed mice (Figure 1A). Restraint-stress increased the size of the adrenal gland by 131%, and decreased the weight of the spleen by 81.5%. THN and HY7715 reduced the mass of the adrenal gland by 77.6% and 76.7%, respectively, compared with that in the stressed group; however, the spleen weight was not recovered by either THN or HY7715, and adrenal weight significantly increased (Figure 1B,C). Moreover, restraint stress decreased the neutral fat and total cholesterol levels from 50.8 mg/dL and 116.0 mg/dL to 23.2 mg/dL and 90.8 mg/dL, respectively, compared with the CON group. By contrast, HY7715 increased these levels to 34.0 mg/dL and 103.6 mg/dL, respectively (Figure 1D,E). In addition, levels of AST and ALT levels, markers of liver damage, also increased after application of restraint stress, to 76.8 U/L and 36.0 U/L, respectively, compared with the normal group (47.4 U/L and 27.8 U/L, respectively). However, HY7715 restored these levels to almost normal (56.7 U/L and 25.7 U/L, respectively; Figure 1F,G). In summary, the data suggest that HY7715 regulates not only impaired lipid metabolism and liver metabolism caused by stress, but also the weight of the adrenal glands.

### 2.2. HY7715 Improves Anxiety-like Behavior in Restraint-Stressed Mice

**Figure 2:**
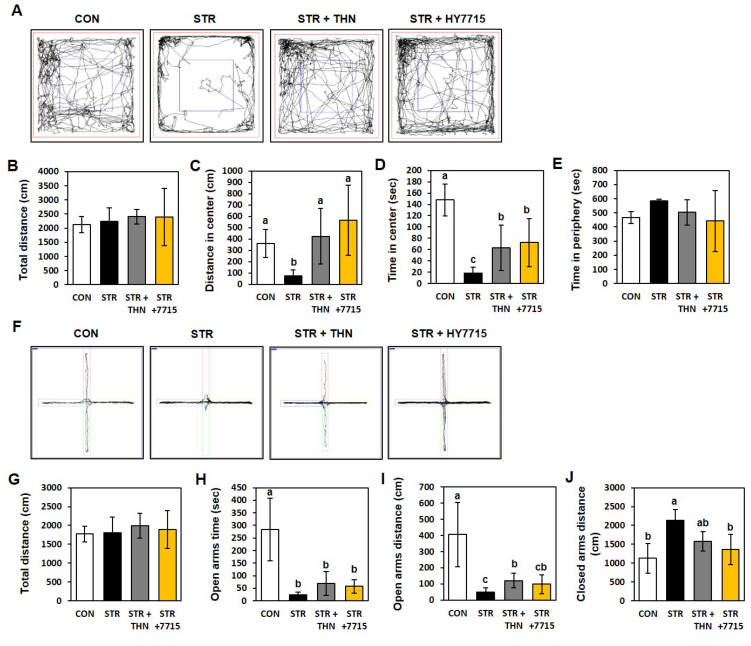
Behavior of restraint-stressed mice in the open field test (OFT) and elevated plus maze (EPM) test. (**A**) Representative tracksheets for the OFT, (**B**) distance traveled in the total zone, (**C**) distance traveled in the central zone, (**D**) time spent in the central zone, and (**E**) time spent in the peripheral zone (Red box, central boundary; Blue box, peripheral boundary). (**F**) Representative tracksheets for the, (**G**) total distance traveled in the arms, (**H**) time spent in the open arm, (**I**) distance traveled in the open arm, and (**J**) distance traveled in the closed arm (*n* = 5) (Red and green, open arms; Blue and yellow, closed arms). Statistics data were analyzed using one-way ANOVA followed by Tukey’s post hoc test. Different letters present significant differences (*p* < 0.05). CTR, control mice; STR, stress-exposed mice; STR + TFN, stress-exposed mice treated with 50 mg/kg/day L-theanine; STRE + 7715, stress-exposed mice treated with HY7715 probiotics (10^8^ CFU/kg/day).

Exploratory behavior, anxiety, and overall activity were measured in the open field test (OFT; Figure 2A). Although there was no significant difference in total distance traveled by all mice, there were differences in the distance and time spent in the center square. Stressed mice showed a reduction in the distance traveled in the center, as well as time spent in the center, by 20.7% and 12.6%, respectively, compared with control mice. However, the mice administered HY7715 showed an increase (757.5% and 390.1%, respectively) in these distances when compared with stressed mice, and the effect was greater than that of THN. By contrast, the time spent in the periphery by stressed mice increased by 125.4% compared with the normal group, and was decreased to 86.1% and 75.7%, respectively, by TFN and HY7715, although the difference was not significant.

Anxiety levels were confirmed in an elevated plus maze (EPM) conducted after restraint stress. Regardless of whether or not the mice were stressed, there was no significant difference in the total distance traveled; however, the time spent and distance traveled in the open arms by stressed mice were only 8.7% and 12.3%, respectively, of that spent by control mice. Mice treated with HY7715 spent significantly more time in the open arms (232.2%, not significant), and traveled a greater distance (196.6%) than stressed mice. Meanwhile, the distance traveled in the closed arms by stressed mice was 189.5% higher than that by the normal group; this was reduced to 63.7% by treatment with HY7715.

### 2.3. HY7715 Regulates BNDF, and mRNA Related to Neuronal Factors in Restraint-Stressed Mice

**Figure 3:**
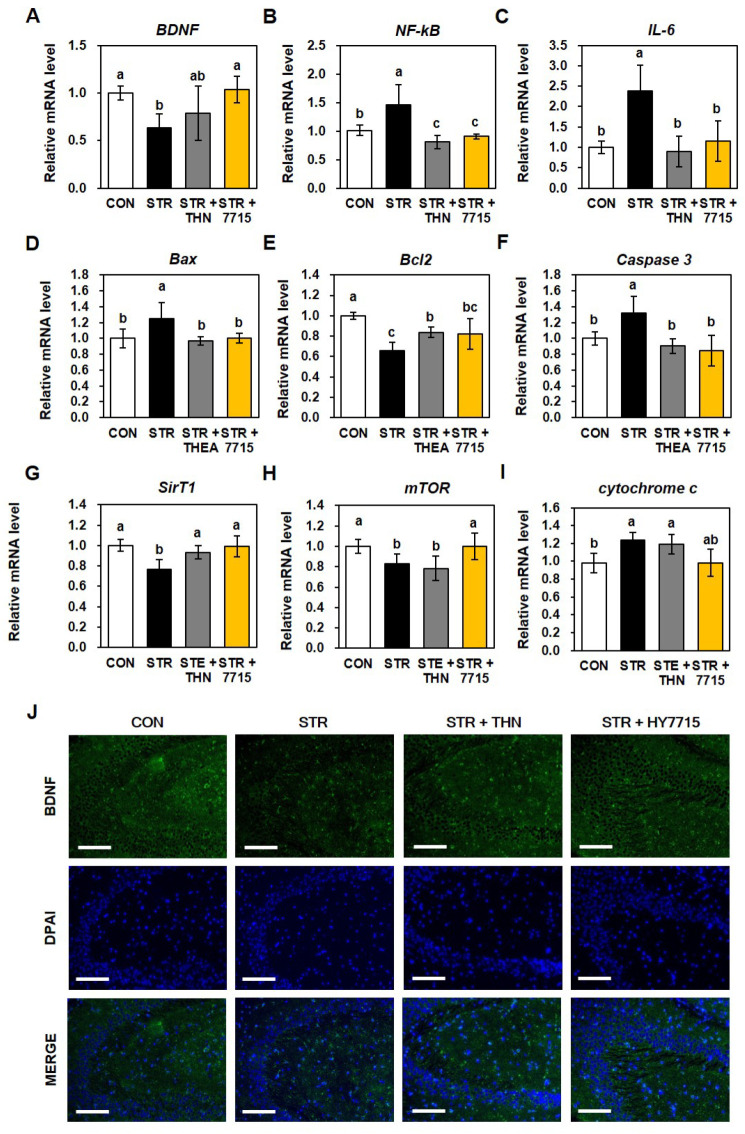
Expression of Brain-Derived Neurotrophic Factor (BNDF) and mRNA related to neuronal factors in restraint-stressed mice. Expression of mRNA encoded by (**A**) BDNF, (**B**) nuclear factor kappa-light-chain-enhancer of activated B cells (NF-κB), (**C**) interleukin 6 (IL-6), (**D**) BCL2 family of proteins (BAX), (**E**) B cell lymphoma 2 protein family (Bcl2), (**F**) caspase 3 (**G**) sirtuin 1 (SIRT1), (**H**) mechanistic target of rapamycin kinase (mTOR) (**I**) cytochrome c (*n* = 4). (**J**) Representative images of immunofluorescence staining for BDNF in mice (scale bar = 100 μm; Green, BNDF expression; Blue, DAPI for nuclei). Statistics data were analyzed using one-way ANOVA followed by Tukey’s post hoc test. Different letters present significant differences (*p* < 0.05). CTR, control mice; STR, stress-exposed mice; STR + TFN, stress-exposed mice treated with 50 mg/kg/day L-theanine; STRE + 7715, stress-exposed mice treated with HY7715 probiotics (10^8^ CFU/kg/day).

We measured levels of brain-derived neurotrophic factor (BDNF) mRNA, inflammatory factors, and antioxidant enzymes in brain tissue from each group. Stress decreased the level of BDNF mRNA by 0.64-fold compared with the normal group. This was increased significantly (to 1.04-fold) by HY7715 (Figure 3A). In addition, stress increased expression of genes encoding inflammatory factors such as nuclear factor kappa-light-chain-enhancer of activated B cells (NF-κB) and interleukin 6 (IL-6) were increased by 1.45-fold and 2.38-fold, respectively. HY7715 reduced these levels to 0.91-fold and 1.16-fold, respectively, similar to levels recorded after treatment with THN (Figure 3B,C). Interestingly, expression of genes associated with apoptosis-related factors BCL2 Associated X (BAX), B cell lymphoma 2 protein family (Bcl2), and caspase 3 was increased significantly (by 1.25-fold, 0.66-fold, and 1.32-fold, respectively) compared with the control group. Intake of HY7715 reduced the increased levels of BAX and caspase 3 significantly, and increased the decreased levels of Bcl2 (Figure 3D–F). In addition, as shown in Figure 3G,H, expression of sirtuin 1 (SIRT1) and mechanistic target of rapamycin kinase (mTOR) in the brains of stressed mice fell by 0.76-fold and 0.83-fold, respectively. However, HY7715 increased expression of both to levels comparable with those in normal mice. Meanwhile, levels of cytochrome c mRNA increased significantly (by 1.23-fold) in response to stress, but were reduced significantly by HY7715 (by 0.98-fold; Figure 3I). Finally, we performed immunofluorescence analysis to detect expression of BDNF protein in mouse brain tissue. Restraint stress reduced BDNF green intensity in hippocampal tissue significantly, which was partially restored by HY7715.

### 2.4. HY7715 Regulates Norepinephrine and Inhibits Pathological Changes in Hippocampus in Restraint-Stressed Mice

To elucidate the role of serum norepinephrine in regulating mood-related behavior, we measured serum norepinephrine, dopamine, cortisol, and serotonin concentrations (Table 1). The level of norepinephrine in stressed mice was 227.6 pg/mL, higher than that in normal mice (158.3 pg/mL). This level in stressed mice was reduced significantly (to 195.9 pg/mL) after treatment with HY7715. We also observed significant differences in dopamine, cortisol, and serotonin levels between stressed and normal mice (Table 1); the levels in stressed mice tended to be restored to normal levels by HY7715 and THN, although the data were not statistically significant.

**Figure 4:**
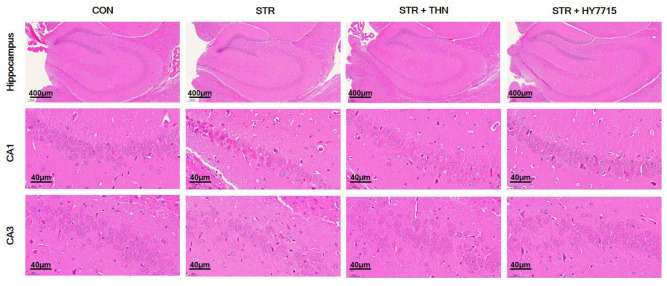
Pathological observation of the hippocampus and cornu ammonis 1 (CA1) and CA3 regions stained with H&E. Hippocampus (scale bar = 400 μm); CA1 and CA3 (scale bar = 400 μm). CTR, control mice; STR, stress-exposed mice; STR + TFN, stress-exposed mice treated with 50 mg/kg/day L-theanine; STRE + 7715, stress-exposed mice treated with HY7715 probiotics (10^8^ CFU/kg/day).

Next, we conducted H&E staining to observe how restraint stress affects the morphological structure of the hippocampus (Figure 4). Restraint stress significantly increased chromatin aggregation and nuclear shrinkage in the CA1 and CA3 regions of the hippocampus, as well as hippocampal neuronal loss. However, HY7715 and THN reduced the stress-induced pathological changes observed in the hippocampus.

### 2.5. HY7715 Protects Neuronal Cells Against H_2_O_2_-Induced Oxidative Stress Damage

**Figure 5:**
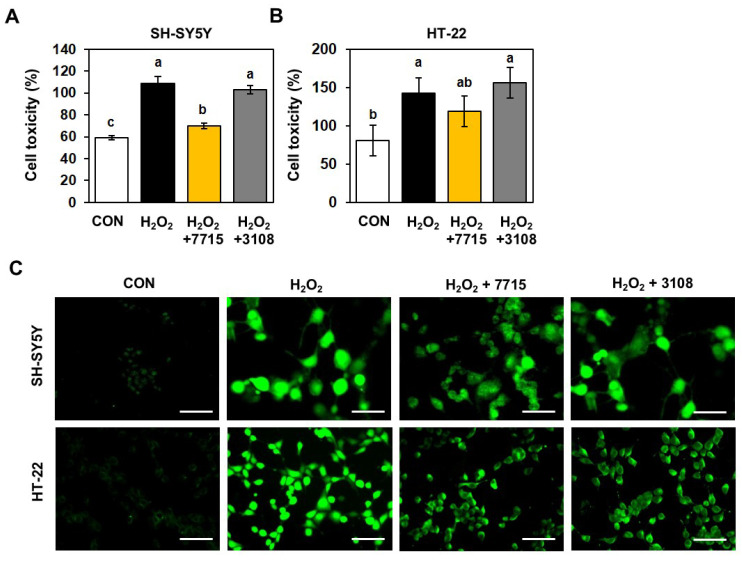
Protective effects of HY7715 against H_2_O_2_-induced cytotoxicity in SH-SY5Y and HT-22 cells. Viability of (**A**) human SH-SY5Y neuroblastoma cells and (**B**) mice neuronal HT-22 cells was measured in a LDH release assay. (**C**) The level of ROS determined by DCFH-DA fluorescence (Green). CON, control cells; H_2_O_2_, H_2_O_2_-exposed cells; H_2_O_2_ + 7715, stress-exposed mice treated with 10^6^ CFU/mL HY7715 probiotics; H_2_O_2_ + 3108; H_2_O_2_-exposed cells treated with 10^6^ CFU/mL of strain type KCTC3108. Scale bar = 100 μm. Statistics data were analyzed using one-way ANOVA followed by Tukey’s post hoc test. Different letters present significant differences (*p* < 0.05).

As shown in Figure 5A, exposure to H_2_O_2_ increased LDH release (109.0%) by SH-SY5Y cells significantly when compared with the amount released by the control group (59.0%). HY7715 inhibited H_2_O_2_-induced cell damage more effectively than the positive control (69.9% and 103.1%, respectively). Furthermore, H_2_O_2_ increased LDH release (142.4%) by HT-22 cells significantly when compared with the amount released by the control group (80.4%) as shown in Figure 5B. Similarly, HY7715 inhibited H_2_O_2_-induced cytotoxicity more effectively than the positive control (118.9% vs. 158.6%, respectively). Next, we used the fluorescent probe DCFH2-DA to determine whether HY7715 prevents H_2_O_2_-induced ROS production (Figure 5C). Intracellular ROS levels in neuronal cells exposed to H_2_O_2_ were significantly higher than those in control cells; however, ROS levels in SH-SY5Y and HT-22 cells pretreated with HY7715 before exposure to H_2_O_2_ were reduced significantly; indeed, the inhibitory effect was greater than that exerted by the strain type KCTC3108. These data suggest that HY7715 protects against cytotoxicity caused by H_2_O_2_-induced oxidative stress, and inhibits ROS production by neuronal cells.

### 2.6. HY7715 Increases Mitochondrial Activity and Protects Neuronal Cells Against H_2_O_2_-Induced Apoptosis

**Figure 6:**
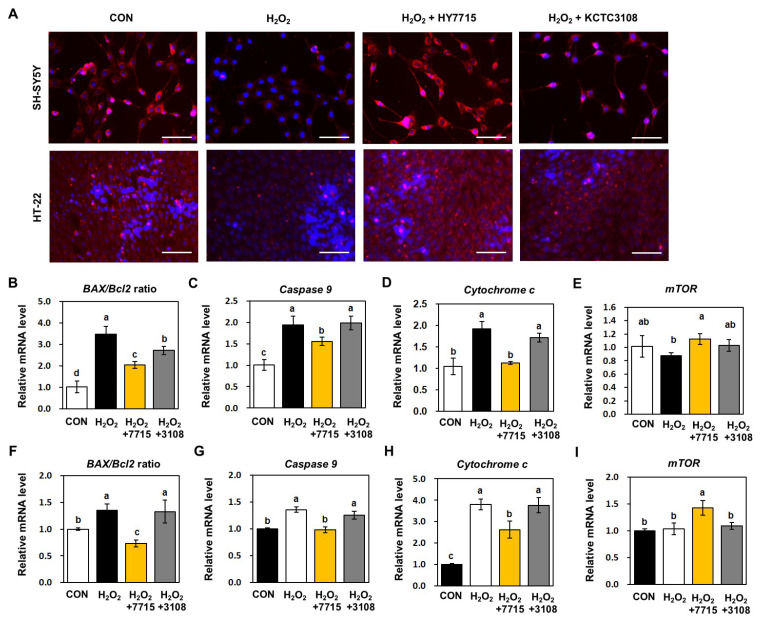
Effects of HY7715 on mitochondrial function and expression of apoptosis-related genes in SH-SY5Y and HT-22 cells. (**A**) Confocal images showing fluorescence signals generated by MitoTracker deep red FM and DAPI; scale bar = 100 μm. Active mitochondria were localized by MitoTracker Red (red); Nuclei were stained with DAPI (blue). Levels of (**B**) BAX/Bcl2 ratio, (**C**) caspase 9, (**D**) mTOR, and (**E**) cytochrome c mRNA in SH-SY5Y cells. The (**F**) BAX/Bcl2 ratio, and levels of (**G**) caspase 9, (**H**) mTOR, and (**I**) cytochrome c mRNA in HT-22 cells. CON, control cells; H_2_O_2_, H_2_O_2_-exposed cells; H_2_O_2_ + 7715, stress-exposed mice treated with 10^6^ CFU/mL HY7715 probiotics; H_2_O_2_ + 3108; H_2_O_2_-exposed cells treated with 10^6^ CFU/mL of strain type KCTC3108. Scale bar = 100 μm. Statistics data were analyzed using one-way ANOVA followed by Tukey’s post hoc test. Different letters present significant differences (*p* < 0.05).

To further demonstrate the ability of HY7715 to protect against oxidative stress, we measured the number of activated mitochondria in SH-SY5Y and HT-22 cells using MitoTracker fluorescence staining. As shown in Figure 6A, the number of intracellular active mitochondria in H_2_O_2_-treated cells was lower than that in control cells. HY7715 restored mitochondrial fluorescence to levels comparable with those in the control group, and was again more effective than KCTC3108. To determine the effect of HY7715 on H_2_O_2_-induced mitochondrial and neuronal cellular damage, we analyzed expression of mRNA encoding BAX/Bcl2, caspase 9, cytochrome c, and mTOR. As shown in Figure 6B–D,F–H, levels of BAX/Bcl2, caspase 9, and cytochrome c mRNA in both neuronal cell lines increased significantly after exposure to H_2_O_2_. Interestingly, HY7715 reduced expression of the three genes significantly. In addition, HY7715 did not affect expression of mTOR in H_2_O_2_-treated cells; however, HY7715 increased expression of mTOR significantly in SH-SY5Y and HT-22 cells (Figure 6E,I). These results suggest that the HY7715 may affect mitochondrial activity and homeostasis, as well as apoptosis induced by oxidative stress.

## 3. Discussion

Probiotics have diverse functional benefits in the host, including regulation of gut immunity, anti-inflammatory effects, and improvement of conditions such as obesity, skin health, metabolic syndrome, muscle strength, and ocular dryness [28,30,31,32,33]. With the recent emergence of psychobiotics, interest in their potential to improve mental health and neurodegenerative disorders has increased [34]. Psychobiotics are live microorganisms that, when administered in adequate amounts, interact with the gut microbiota to modulate brain function and mental health [35]. For example, clinical evidence shows that certain Bifidobacterium strains alleviate symptoms of stress and depression, although most studies relied on subjective assessments rather than elucidating the underlying molecular mechanisms [36]. This highlights the need for mechanistic studies to clarify how psychobiotics exert neuroprotective effects.

Anxiety disorders are multifactorial, with chronic and repetitive stress being a primary trigger. Beyond psychological distress, stress contributes to a range of physical symptoms and increases the risk of mental illness [37]. Chronic stress disrupts neural function, leading to impaired cognition, mood regulation, and sleep, and can alter the composition of the gut microbiota, thereby affecting behavior via the gut–brain axis. Therefore, research on the use of probiotics, including LAB, for stress relief has attracted much attention [38,39]. HY7715, isolated from kimchi, is a bile acid-tolerant LAB with an established safety profile for fermentation applications [27]. However, its psychobiotic potential as a treatment for stress-induced behavioral and neurophysiological changes has not been investigated. In this study, to determine whether HY7715 improves stress-induced anxiety-like behavior, we administered HY7715 (10^9^ CFU/kg/day of) orally to mice for 3 weeks, and then exposed them to restraint stress (2 h/day for 14 days). Additionally, we used L-theanine as a positive control, which has been shown to reduce stress and enhance cognitive function. This is a natural amino acid analog found in green tea and has been reported to regulate neurotransmitter levels and alleviate anxiety [40,41]. Moreover, we investigated the neuroprotective effects of HY7715 in HT-22 mouse hippocampal neuroblast cell and SH-SY5Y human neuroblastoma cells.

To determine whether HY7715 probiotics can overcome anxiety-like behaviors induced by restraint stress, we measured anxiety levels and despair scores in the OFT and EPM. We found that restraint stress reduced exploratory behavior and increased anxiety indices, and that HY7715 restored open-arm activity and central exploration significantly without affecting total locomotion. These results suggest that HY7715 alleviates stress-induced anxiety-like behaviors through a neuroprotective mechanism. Furthermore, we investigated the effects of restraint stress on the body weight and various physiological and biochemical markers in the blood of mice. Physiologically, restraint stress induced anorexia-like weight loss, altered lipid metabolism, and increased hepatic stress markers. Although HY7715 did not reverse weight loss, it restored triglyceride and cholesterol levels and normalized ALT and AST levels, suggesting systemic protective effects associated with chronic stress and related neurobehavioral changes [42]. Such metabolic and hepatic improvements may contribute, directly or indirectly, to enhanced brain function and stress resilience. HY7715 also reduced adrenal hypertrophy, an indicator of HPA axis hyperactivation, further supporting its role in stress adaptation. Previous studies show that excessive stress-induced alterations in adrenal weight and function can lead to anxiety, chronic fatigue, impaired concentration, indigestion, and muscle pain [43,44].

In addition, restraint stress elevated norepinephrine, dopamine, and cortisol levels while reducing serotonin levels. HY7715 normalized these changes, at least partially, by reducing norepinephrine, a key mediator of ROS generation and neuroinflammation. This indicates that HY7715 can alleviate stress-induced hormonal imbalances, suggesting that it may be involved in the HPA axis interaction. Histological analysis revealed that restraint stress caused neuronal damage to the CA1 and CA3 hippocampal regions, including chromatin condensation and reduced cell density, which were improved markedly by HY7715 treatment. Hippocampal structure can be altered by excessive stress responses, which can lead to cognitive dysfunction; therefore, HY7715 may improve anxious behavior by preventing structural changes in the hippocampus caused by excessive restraint stress.

BDNF plays a role in brain inflammatory processes, and decreased BDNF levels can lead to brain dysfunction [45]. Furthermore, models of anxiety show that BDNF and apoptosis are closely related [46,47]. In particular, the balance between BAX and Bcl2 plays a crucial role in regulating apoptosis, and caspase-3 activation is essential for neuronal apoptosis [48]. At the molecular level, restraint stress suppressed BDNF expression and upregulated pro-inflammatory markers (NF-κB, IL-6), pro-apoptotic signaling (BAX/Bcl-2 ratio, caspase-3), and markers of mitochondrial dysfunction (i.e., cytochrome c release). HY7715 restored BDNF levels, reduced neuroinflammation, normalized apoptotic markers, and increased expression of mitochondrial regulatory genes (SIRT1, mTOR), suggesting coordinated protection against oxidative stress-mediated neuronal injury. Recent studies report that maintaining mitochondrial function is essential for preventing acute and chronic brain damage [49]. Dysfunction of mitochondrial bioenergetics leads to inefficient production of ROS, inadequate energy supply via ATP, and abnormal release of mitochondrial cytochrome c [50]. One of the key factors that induces apoptosis is release of cytochrome c from mitochondria [51]. By contrast, SIRT1, an NAD-dependent deacetylase, can enhance mitochondrial function and reduce oxidative stress [52]. SIRT1 is associated with age-related ROS production, which is heavily dependent on mitochondrial metabolism [53]. Furthermore, mTOR activation prevents neuronal loss, blocks oxidative stress-induced microglial damage, and protects against neuronal damage and neurodegenerative diseases [54]. Furthermore, additional regulatory pathways may also be involved. The aryl hydrocarbon receptor (AhR) and peroxisome proliferator-activated receptor gamma (PPARγ) are closely connected with SIRT1 and mTOR signaling. Both receptors are known to modulate inflammatory and apoptotic processes in the central nervous system [55,56]. In particular, AhR has emerged as a key mediator of gut–nervous system interactions by sensing microbial metabolites such as indoles and tryptophan derivatives [57]. PPARγ, on the other hand, exerts anti-inflammatory effects through cross-talk with NF-κB and mitochondrial pathways [58]. Considering these interactions, it is plausible that HY7715 may indirectly influence AhR and PPARγ signaling via modulation of oxidative stress and mitochondrial function. Future studies are warranted to clarify whether these receptors contribute to the psychobiotic effects of HY7715.

To better understand the molecular cellular mechanism underlying oxidative stress, we induced oxidative stress in SH-SY5Y and HT-22 neuronal cell lines by exposing them to H_2_O_2_, and then compared the differences between HY7715 and the control, KCTC3108. Excessive ROS production induced by H_2_O_2_ led to increased oxidative stress, mitochondrial DNA damage, neuronal energy metabolism disorders, and decreased cell viability. HY7715 demonstrated superior antioxidant and mitochondrial-protective effects in H_2_O_2_-induced oxidative stressed SH-SY5Y and HT-22 cells than the reference strain KCTC3108. HY7715 reduced H_2_O_2_-induced ROS accumulation, preserved mitochondrial activity, and prevented apoptosis by modulating expression of BAX/Bcl-2, caspase-9, and mitochondrial genes, all of which were consistent with the in vivo findings. In this study, only male mice were used, and the behavioral assessments were restricted to anxiety-related paradigms without evaluation of depression-specific models. Because females have estrous cycles and associated hormonal fluctuations, male mice were used in this study to reduce hormonal variability [59,60]. Despite limitations such as the need for additional research and future studies to confirm the effects of HY7715, our findings provide important evidence that *Lactiplantibacillus plantarum* HY7715 exerts beneficial effects on stress-related physiology and anxiety-like behaviors, supporting its potential as a psychobiotic candidate.

## 4. Materials and Methods

### 4.1. Culture of Lactic Aicd Bacteria

*Lactobacillus plantarum* HY7715, isolated from Korean kimchi, was provided by hy Co., Ltd. *L. plantarum* HY7715 was cultured in de Man Rogosa Sharp broth (Difco Corp., Sparks, MD, USA) at 37 °C for 15–20 h. Cultured HY7715 cells were centrifuged at 3000× *g* for 15 min, and the supernatants discarded. The cell pellets were washed twice with sterile phosphate-buffered saline (PBS) and resuspended in PBS. The concentration of HY7715 was adjusted to 1 × 10^9^ CFU in PBS of 200 μL prior to administration to mice.

### 4.2. Animals and Experimental Design

Male C57BL/6N mice (7 weeks old) were purchased from DBL (Chungcheong-do, Republic of Korea). Mice were acclimated for 1 week in a controlled environment under a 12 h light/dark cycle, a temperature of 19–25 °C, and a relative humidity of 30–70%. During this period, the mice were permitted free access to water and food (Teklad Certified Irradiated Global 18% Protein Rodent Diet 2018C; Envigo RMS, Inc., Indianapolis, IN, USA). After the acclimation period, mice were assigned randomly to one of four groups (*n* = 6/group): CON (non-restraint stress + saline); STR (restraint stress + saline); STR + 7715 (restraint stress + *L. plantarum* HY7715 at 1 × 10^9^ CFU/kg/day); or STR + THN (restraint stress + L-theanine at 50 mg/kg/day, positive control). The CON and STR groups received saline, while the STR + 7715 and the STR + THN groups were orally administered HY7715 or L-theanine once daily for 21 days. After 14 days, restraint stress was applied by placing the mice in a restrainer for 2 h daily for 7 days. Saline, STR, or THN was administered 1 h before application of restraint stress. Body weight was measured every other day during the 14-day treatment period using a calibrated digital balance. Measurements were performed at the same time in the morning, prior to restraint stress exposure, to minimize variability caused by transient stress-induced weight changes. On Day 6 after restraint stress was exerted, the mice were subjected to an OFT. On Day 7, mice were subjected to an EPM. All behavioral tests were evaluated 30 min after restraint stress. At the end of the experimental period, the mice were fasted for 12 h and euthanized in a CO_2_ gas chamber. Immediately after euthanasia, blood samples were collected from the inferior vena cava using a 35 G needle, and serum was separated by centrifugation for subsequent biochemical and hormonal analyses.

### 4.3. OFT

The OFT evaluates locomotor activity and willingness to explore. Mice were placed in a white acrylic apparatus (41 cm × 41 cm × 40 cm) with a square (20.5 cm × 20.5 cm) marked by black lines in the center. Mice were allowed to explore the center of the box freely for 10 min, and behaviors were recorded using SMART 3.0 Video Tracking Software (Panlab, Harvard Apparatus, Barcelona, Spain). The total distance traveled, the distance traveled within the center, and the time spent in the center or periphery, were analyzed.

### 4.4. EPM

The EPM is a common behavioral test used to define the underlying mechanism of anxiety-related behavior in rodents. Behavior in a white acrylic maze consisting of two open and closed arms (all arms: 76 cm × 5 cm) at a height of 50 cm from the floor was assessed. Each mouse was in the center square (5 cm × 5 cm) facing the arms, and allowed to explore for 10 min. Behaviors were recorded with SMART 3.0 Video Tracking Software. The total distance traveled, the distance traveled in the open or closed arms, the time spent in the open arms, and the percentage of time spent in the open arms were analyzed.

### 4.5. Analysis of Biochemical Parameters

Blood samples were centrifuged for 15 min at 3000× *g* to separate the serum. The serum level of liver function markers such as AST (ab263882, Abcam, Cambridge, UK) and ALT (ab282882) was measured. In addition, markers of energy metabolism (creatine kinase and creatine) were evaluated. Furthermore, biomarkers of lipid metabolism (triglyceride (MBS168769, MyBioSource, San Diego, CA, USA), total cholesterol (MBS269999)) were assessed using commercial ELISA kits.

### 4.6. Analysis of Serum Hormone Levels

Serum levels of norepinephrine, dopamine, cortisol, serotonin, and stress-related hormones were quantified using ELISA kits (MyBioSource, San Diego, CA, USA; MBS2097837, MBS9718203, MBS704879, MBS2611552). The neurotransmitter norepinephrine (#MBS1603771) was also evaluated.

### 4.7. qRT-PCR

Samples were homogenized using TRIzol reagent and total RNA extracted using an Easy-spin Total RNA Extract Kit (iNtRON Biotechnology, Seoul, Republic of Korea). Next, cDNA was synthesized using an Omniscript Reverse Transcription Kit (Qiagen, Hilden, Germany). RT-qPCR (Applied Biosystems, Carlsbad, CA, USA) was performed using the TaqMan^TM^ Gene Expression Assay (Applied Biosystems). Quantification of *BDNF* (Mm04230607_s1), *NF-κB* (Mm00476361_m1), *IL-6* (Mm01211445_m1), *Bax* (Mm00432051_m1, Hs00180269_m1), *Bcl2* (Mm00477631_m1, Hs04986394_s1), *Caspase 3* (Mm01195085_m1), *Caspase 9* (Hs00962278_m1), *SirT1* (Mm01168521_m1), *mTOR* (Mm00444968_m1, Hs00234508_m1), *cytochrome c* (Mm01621048_s1, Hs01588974_g1), and *GAPDH* (Mm99999915_g1, Hs02786624_g1) transcripts was conducted using gene-specific primers. Relative mRNA levels were calculated using the 2^(−ΔΔCT)^ method, and the expression normalized to that of *GAPDH*.

### 4.8. Histological Analysis

H&E staining was performed to analyze the histological structure of the hippocampus. Briefly, the hippocampus was fixed in 4% paraformaldehyde at room temperature for 24 h and then embedded in paraffin. Paraffin blocks were sectioned at a thickness of 4 μm and stained with H&E. Overall morphology, as well as that of the CA1 and CA3 regions of the hippocampus, was observed under a microscope (Nikon Eclipse E600; Nikon Corporation, Tokyo, Japan).

### 4.9. Immunofluorescence Staining

Immunofluorescence staining was performed to visualize expression of BDNF in the hippocampus. Briefly, tissue sections were washed three times at room temperature with Tris-buffered saline (TBS, EBA-1101, Elpisbio, Daejeon, Republic of Korea). Antigen retrieval was conducted using Target Retrieval Solution Citrate, pH 6.0 (S2369, DAKO, Agilent Scientific Instruments, Santa Clara, CA, USA), followed by blocking at 37 °C for 2 h in PBS containing 10% goat serum (G9023, Sigma-Aldrich, St. Louis, MO, USA) and 0.3% Triton X-100 (X100, Sigma-Aldrich, USA) to minimize nonspecific binding. After blocking, sections were incubated overnight at 4 °C with an anti-BDNF antibody (1:500, ab108319, Abcam, Cambridge, UK). Then, sections were washed three times in TBS and incubated at room temperature for 1 h in the dark with a fluorescent secondary antibody (Alexa Fluor 488; 1:500; ab150077, Abcam, Cambridge, UK). After 5 min of nuclear staining with DAPI (ab104139, Abcam, Cambridge, UK), sections were rinsed with distilled water, covered using a fluorescence-compatible mounting medium, and cover-slipped. Fluorescence images were observed using a ZOE Fluorescent Cell Imager (BioRad, Hercules, CA, USA).

### 4.10. Neuronal Cell Culture Conditions

SH-SY5Y human neuroblastoma cells were obtained from the Korean Cell Line Bank (Seoul, Republic of Korea), and HT-22 mouse hippocampal neuroblast cells were obtained from American Type Culture Collection (ATCC; Manassas, VA, USA). SH-SY5Y cells were grown in Minimum Essential Medium (MEM; Welgene, Gyeongsan-si, Gyeongsangbuk-do, Republic of Korea) containing 10% fecal bovine serum (FBS; Gibco, Grand Island, NY, USA), 1% penicillin-streptomycin (Gibco), and 25 mM HEPES (Gibco). Differentiation of SH-SY5Y cells was induced for 5 days by replacing the medium with MEM supplemented with 2% FBS and 10 μM retinoic acid (Sigma-Aldrich).

HT-22 cells were grown in Dulbecco’s modified Eagle’s Medium (Gibco) supplemented with 10% FBS (Gibco) and 1% penicillin-streptomycin (Gibco). These cells were incubated at 37 °C in a humidified atmosphere containing 5% CO_2_.

### 4.11. LDH Test

The protective effect against H_2_O_2_-induced oxidative damage was studied using retinoic acid-differentiated (neuron-like) human SH-SY5Y cells and hippocampal neuroblast HT-22 cells. SH-SY5Y and HT-22 cells were seeded into 96-well plate at a density of 1 × 10^5^ cells/well and 5 × 10^4^ cells/well, respectively, and then cultured overnight. Cells were pretreated with HY7715 (10^6^ CFU/mL) for 6 h, followed by co-treatment with H_2_O_2_. SH-SY5Y cells were treated with 400 μM H_2_O_2_ for 24 h, and HT-22 cells were treated with 200 μM H_2_O_2_ for 24 h, and then analyzed using the LDH assay. The supernatants were collected and released LDH was measured using a CytoTox 96^®^ Non-Radioactive Cytotoxicity Assay Kit (Promega; Wisconsin, Madison, WI, USA).

### 4.12. Measurement of ROS

Intracellular ROS levels were evaluated using 2′, 7′-dichlorofluorescein diacetate (DCFH-DA; Sigma-Aldrich). Cells were prepared and treated with HY7715 and H_2_O_2_ as described for the LDH assay. Afterwards, cells were washed twice with PBS, followed by incubation at 37 °C for 30 min in the dark in serum-free medium containing 10 μM of DCFH-DA. Intracellular ROS levels were measured using a BioTek^®^ Synergy HTX multimode reader (Agilent Technologies, Santa Clara, CA, USA).

### 4.13. Mitotracker Deep Red FM Staining

A commercial MitoTracker staining method was used to visualize mitochondrial morphology. Cells were incubated at 37 °C for 20 min in serum-free medium containing 500 nM of MitoTracker Deep Red FM (Cells Signaling, Danvers, MA, USA). The cells were then washed twice with FBS and visualized using a ZOE Fluorescent Cell Imager (BioRad).

### 4.14. Statistical Analysis

Statistical data were analyzed by using SPSS (version 20.0, IBM, Inc., Armonk, NY, USA). Data values are expressed using the mean ± standard deviation. They were compared through one-way ANOVA, followed by Tukey’s test. *p* < 0.05 was considered to indicate statistical significance.

## 5. Conclusions

This study demonstrates that *Lactiplantibacillus plantarum* HY7715 effectively alleviates restraint stress-induced anxiety-like behaviors in mice. HY7715 restored hippocampal structure, normalized stress-related neurochemical changes, and suppressed neuroinflammation. Mechanistically, it reduced oxidative stress, inhibited apoptosis, and preserved mitochondrial function both in vivo and in vitro. These findings suggest that HY7715 is a promising psychobiotic candidate that prevents stress-related neuropsychiatric disorders by modulating neuronal oxidative and apoptotic pathways.

## Figures and Tables

**Table 1:** Serum hormone levels in restraint-stressed mice.

Serum Hormones	CON	STR	STR + THN	STR + 7715
Norepinephrine (pg/mL)	158.3 ± 6.5 ^b^	227.6 ± 23.8 ^a^	198.6 ± 13.7 ^b^	195.9 ± 7.9 ^ab^
Dopamine (pg/mL)	852.0 ± 188.9 ^b^	18029 ± 35.9 ^a^	1648.1 ± 130.8 ^a^	1504.0 ± 381.3 ^a^
Cortisol (ng/mL)	135.0 ± 3.7 ^b^	166.7 ± 6.8 ^a^	145.0 ± 20.5 ^b^	149.6 ± 13.4 ^b^
Serotonin (pg/mL)	37.2 ± 2.8 ^a^	29.1 ± 2.1 ^b^	32.1 ± 1.4 ^a^	30.0 ± 4.0 ^a^

Statistics data were analyzed using one-way ANOVA followed by Tukey’s post hoc test. Different letters present significant differences (*p* < 0.05).

## Data Availability

The original contributions presented in this study are included in the article. Further inquiries can be directed to the corresponding author.

## Abbreviations

The following abbreviations are used in this manuscript:

ALT	alanine aminotransferase
AST	aspartate aminotransferase
BAX	BCL2 Associated X
Bcl2	B cell lymphoma 2 protein family
BDNF	brain-derived neurotrophic factor
CA	cornu ammonis
CK	creatine kinase
CON	non-restraint stress + saline
DCFH-DA	2′,7′-dichlorofluorescein diacetate
DMEM	Dulbecco’s modified Eagle’s Medium
EPM	elevated plus maze test
H&E	hematoxylin-eosin
HDL	high-density lipoprotein
HPA	hypothalamic–pituitary–adrenal
HY7715	*Lactiplantibacillus plantarum* HY7715
IF	immunofluorescence
IL-6	interleukin-6
LAB	lactic acid bacteria
LDH	lactate dehydrogenase
LDL	low-density lipoprotein
MEM	Minimum Essential Medium
mTOR	mechanistic target of rapamycin kinase
NFkB	nuclear factor kappa-light-chain-enhancer of activated B cells
OFT	open field test
PBS	phosphate-buffered saline
ROS	reactive oxygen species
SirT1	sirtuin 1
STR	restraint stress + saline
STR + 7715	restraint stress + *L. plantarum* HY7715 at 1 × 10^9^ CFU/kg/day
